# Transcriptome‐Wide Association Analysis of Flavonoid Biosynthesis Genes and Their Correlation With Leaf Phenotypes in Hawk Tea ( *Litsea coreana* var. *sinensis* )

**DOI:** 10.1002/ece3.70563

**Published:** 2024-11-17

**Authors:** Lan Yang, Huie Li, Na Xie, Gangyi Yuan, Qiqiang Guo

**Affiliations:** ^1^ Institute for Forest Resources and Environment of Guizhou, Key Laboratory of Forest Cultivation in Plateau Mountain of Guizhou Province, College of Forestry Guizhou University Guiyang People's Republic of China; ^2^ College of Agriculture Guizhou University Guiyang People's Republic of China; ^3^ The People's Government of Yongshan County Zhaotong Yunnan People's Republic of China

**Keywords:** antioxidant compound, GWAS, SNP, structural genes

## Abstract

Hawk tea (*Litsea coreana* var. *sinensis*), derived from the tender shoots or leaves, rich in flavonoids can promote healthcare for humans. The primary flavonoid are kaempferol‐3‐O‐*β*‐D‐glucoside, kaempferol‐3‐O‐*β*‐D‐galactoside, quercetin‐3‐O‐*β*‐D‐glucoside, and quercetin‐3‐O‐*β*‐D‐galactoside. The existence of an association between leaf phenotype and flavonoid content, along with the underlying mechanisms of flavonoid biosynthesis, remains incompletely understood. In this study, 109 samples were analyzed to determine the correlation and genetic variability in leaf phenotype and flavonoid content. Furthermore, a transcriptome‐wide association study identified candidate loci implicated in the biosynthesis of four key flavonoids. The study revealed that genetic variability in leaf traits and flavonoid concentrations is predominantly attributed to interpopulation differences. Flavonoid accumulation was significantly correlated with tree DBH, indicative of age‐related traits. Transcriptome‐wide association analysis identified 84 significant SNPs associated with flavonoid content, with only 13 located within gene regions. The majority of these genes are implicated in metabolic processes and secondary metabolite biosynthesis. Notably, structural genes within these regions are directly involved in pathways known to regulate flavonoid metabolism, exerting a pivotal influence on flavonoid biosynthesis. These results revealed the physiological basis for the regulation of flavonoid content, as well as the molecular mechanisms for the biosynthesis of flavonoids in hawk tea. It also lays theoretical groundwork for subsequent explorations into the genetic determinants influencing flavonoid accumulation of hawk tea.

## Graphical abstract

A total of 84 significant SNPs related to flavonoid content were identified through transcriptome‐wide association analysis, and 13 SNPs located within gene regions were identified as structural genes primarily implicated in metabolic processes and the biosynthesis of secondary metabolites.

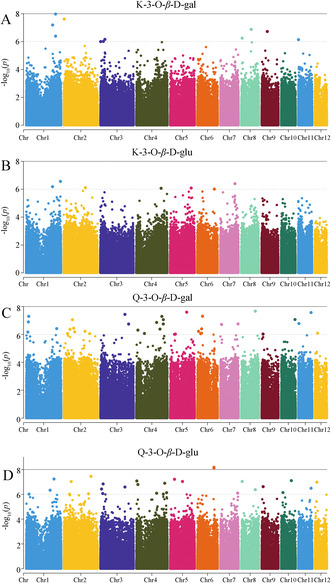

## 1 Introduction

Hawk tea (*Litsea coreana* var. *sinensis*), an ancient tea species endemic to China, has been cultivated and consumed for millennia in the southwest region (Jia et al. 2017). The tea is primarily derived from tender shoots and leaves, rich in flavonoids, amino acids, volatile oils, and other bioactive compounds (Ye et al. 2012). Research has highlighted that hawk tea's predominant polyphenols are flavonol glycosides, distinguishing it as a caffeine‐free beverage (Liang et al. 2007). Flavonols, a subset of flavonoids characterized by a hydroxyl flavone backbone, vary due to the phenolic hydroxyl groups' substitution patterns (Singh et al. 2013). Among the most prevalent flavonoids in vegetation, quercetin and kaempferol stand out as hawk tea's principal flavonols, undergoing glycosylation predominantly at the carbon ring's Position 3 (Liu et al. 2020). Glycosylation of flavonols plays a dual role in plants, it not only confers a yellow hue to plant tissues but also serves as accessory pigment (Zhao et al. 2024). The glycosylation at the third position of the carbon ring in flavonoids is primarily aimed at enhancing their solubility within the aqueous phase (Kazuma, Noda, and Suzuki 2003). This modification is crucial as it allows for efficient transport and bioavailability of these compounds within the plant system, thereby optimizing their physiological functions and contributing to the overall pigmentation and stress response mechanisms (Naing and Kim 2021). In addition to their plant‐based roles, flavonol glycosides exhibit significant antioxidative activities and stability against light, heat, and oxygen, offering the potential to scavenge free radicals (Fan et al. 2022), inhibit oxidase activity, and provide preventive benefits against cardiovascular, cerebrovascular diseases, and cancer (Bondonno et al. 2019). Their antioxidative properties are intricately linked to antiaging, with flavonol glycosides playing a crucial role in delaying aging processes, protecting against Alzheimer disease, and boosting immunity (Yao et al. 2004). In an era marked by growing chronic disease prevalence and a booming food industry, the focus on food health and safety has intensified, spotlighting the development of green health foods and natural additives (Carmela and Giovanna 2022). Hawk tea's inherent health benefits and natural properties underscore its promising future in the food sector.

Current research on hawk tea primarily concentrates on the isolation and characterization of its flavonoid compounds and its pharmacological properties (Jia et al. 2017). The flavonoid content has emerged as a critical parameter for assessing the quality of hawk tea germplasm resources. Investigations have revealed significant variability in leaf morphology across different germplasm resources of the same species, serving as a potential criterion for germplasm identification (Khan et al. 2018). This variability may also, to some extent, indicate differences in flavonoid content among these resources (Song et al. 2022). Previous research has uncovered the composition of the main flavonol components in hawk tea, predominantly consisting of kaempferol‐3‐O‐*β*‐D‐glucoside (K‐3‐O‐*β*‐D‐glu), kaempferol‐3‐O‐*β*‐D‐galactoside (K‐3‐O‐*β*‐D‐gal), quercetin‐3‐O‐*β*‐D‐galactoside (Q‐3‐O‐*β*‐D‐gal), and quercetin‐3‐O‐*β*‐D‐glucoside (Q‐3‐O‐*β*‐D‐glu) (Tan et al. 2016). Recent research offers scant insights into whether leaf morphological traits in hawk tea germplasm resources serve as indicators of flavonoid content. Additionally, diameter at breast height (DBH) has been proposed by Wu et al. (2019) as a growth attribute for identifying superior hawk tea germplasm, particularly when flavonoid content is the primary trait of interest.

Association analysis aims to identify quantitative trait loci through the linkage disequilibrium between different alleles on chromosomes (Liao et al. 2021). A genome‐wide association study (GWAS) can serve as a method to investigate genes associated with quantitative traits (e.g., flavonols) in hawk tea. GWAS employs a vast array of high‐density single nucleotide polymorphisms (SNPs) throughout the genome as molecular genetic markers for conducting genome‐wide correlation analyses (Bhinder et al. 2022). This involves assessing the correlation significance between each variant locus and the target trait, thereby identifying specific gene locus variations that influence the complex trait (Li et al. 2018). A significant advantage of GWAS lies in the ability to utilize the same genotyping data and population samples for investigating various traits (Fang et al. 2021). However, it is essential to recognize that genomes are dynamic entities at the species level, with the genome of an individual representing merely a minute segment of the total genomic diversity within a population (Kong et al. 2022). While de novo genome assembly for highly heterozygous species, such as forest trees, is both labor intensive and costly, transcriptome analysis presents a more accessible and cost‐effective approach (Wu et al. 2024). This method allows for the exploration of genetic variation in population traits at the transcriptional level, particularly for nonmodel plants that may not have a reference genome available. The complete genome of hawk tea has not been published yet, and possessing a reference genome is a fundamental prerequisite for GWAS analysis (Luo et al. 2019). The continuous advancements in transcriptome sequencing technology coupled with decreasing sequencing costs have facilitated the development of transcriptome‐wide association analysis methods. These methods are particularly suited for species whose genomes have not yet been sequenced (Maeda et al. 2019). Utilizing transcriptome sequencing (mRNA‐Seq) data to derive gene expression or structural variations and their correlation with phenotypic variations was initially implemented in *Brassica chinensis* (Harper et al. 2012). Compared to GWAS, transcriptome‐wide association analysis can identify new candidate genes that, upon functional validation, are capable of regulating target traits, thus demonstrating the reliability of the results obtained through this method (Kim et al. 2011). Given that the full genome data for hawk tea remains unpublished, full‐length transcriptome sequencing has become increasingly significant for this species.

Revealing the association between flavonoid content and leaf phenotype is the crucial link for breeding germplasm resources with high flavonoid content in hawk tea. And exploring the genetic mechanism of flavonoid biosynthesis and mining the key genetic loci filled the gap in the research on the mechanism of flavonoid biosynthesis in this species at the population level. Therefore, in this study, the genetic and phenotypic differentiation coefficients of leaf character, DBH, and flavonoid content of one leaf and two buds in 109 samples of hawk tea from five regions were calculated and correlation analysis was conducted. Furthermore, transcriptome sequencing was conducted on 109 samples, utilizing second‐ and third‐generation sequencing technologies. Subsequently, transcriptome‐wide association analysis was conducted, leveraging data on flavonoid content and a high‐quality SNP dataset. The aim of the study was to investigate whether the variation in leaf character, DBH, and flavonoid content in hawk tea primarily originates between or within populations, identify variables highly associated with flavonoid content, and ascertain SNPs with high correlations to flavonoid biosynthesis in hawk tea. In practical applications, the selection of superior traits within the hawk tea species is a matter of great urgency. Therefore, the exploration of genetic loci linked to these crucial traits is deemed of substantial importance. Consequently, the inaugural transcriptome‐wide association analysis in hawk tea has been undertaken in this study, with the objective of identifying significant SNPs associated with the four flavonoids. Results will provide theoretical guidance for understanding the physiological growth, stress response, and other important biological processes, and also provide important technical support for genetic improvement and molecular‐assisted breeding of hawk tea.

## 2 Materials and Methods

### 2.1 Leaf Character, DBH, and Flavonoid Content Determination and Analysis

**FIGURE 1:**
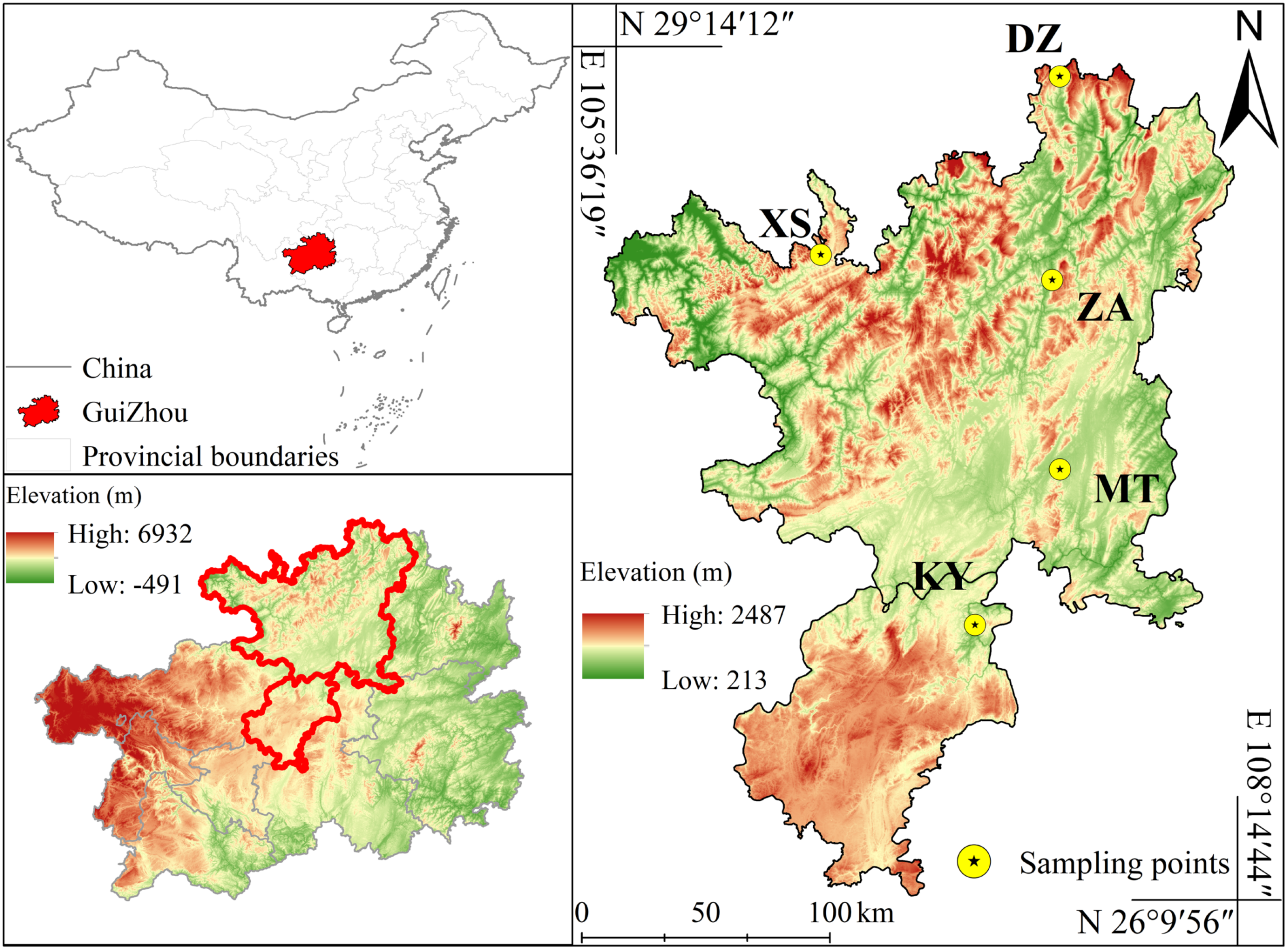
A map showing the natural distribution and the location of study areas in Guizhou Province, SW China (KY: Kaiyang County, XS: Xishui County, MT: Meitan County, DZ: Daozhen County, and ZA: Zheng'an County).

Hawk tea is classified as a diploid organism (Ha et al. 2022). In May 2021, samples of the same species were collected from five sites in Kaiyang County (KY), Xishui County (XS), Meitan County (MT), Daozhen County (DZ), and Zheng'an County (ZA) in Guizhou Province, China (Figure 1). The five sites feature a subtropical humid monsoon climate, characterized by distinct local microclimates and significant vertical climate variations. The average annual temperature ranges from 13.19°C to 15.59°C, with annual precipitation between 1,080 and 1,255 mm (Yuan et al. 2023). Hawk tea was systematically investigated and sampled at the designated site, with adult plants being specifically targeted for sampling. To mitigate the impact of kinship relations, a minimum distance of 30 m was maintained between each sampled individual. One hundred and nine samples were collected in total, including 21 samples from DZ County; they were primarily found in open areas near the river and on the hillside, with limited seedling regeneration, the slope ranged from 7° to 18°, facing southeast. Twenty‐two samples from XS County were primarily distributed in evergreen broadleaved forests surrounding cultivated land and on nearby slopes; seedling regeneration is observed under the forest canopy, with slopes ranging from 10° to 15° and facing southwest. Nineteen samples from ZA County were primarily found in secondary evergreen broad‐leaved forests or bamboo forests with high canopy density, adult individuals are few and mostly located in open areas, and the slope ranges from 3° to 8° and faces southwest. Twenty samples from KY County were primarily found in mountain orchards near villages, characterized by a low canopy and slopes ranging from 8° to 15°, facing southwest. And 27 samples from MT County were primarily distributed in open mountains near cultivated land and around the reservoir, no seedling regeneration was observed, and the slopes range from 8° to 16° and face south.

The DBH of each tree was recorded, and mature leaf samples free from pests and diseases were individually collected from the cardinal directions—southeast and northwest. Following labeling, the samples were secured in ziplock bags, stored at 4°C in a portable refrigerator, and transported to the laboratory on the same day for assessment of leaf phenotypic indicators. In addition, one leaf and two buds were collected, wrapped carefully in tin foil, labeled, immediately frozen in liquid nitrogen, and stored in a –80°C refrigerator for further analysis.

Leaf length (LL), leaf width (LW), leaf area (LA), leaf thickness (LT), and leaf perimeter (LP) were quantified using a portable LA meter (AM350, ADC, UK), and the leaf shape index (LS) (LL/LW) was calculated. Leaf petiole length (LPL) was measured with an electronic digital caliper to an accuracy of 0.01 mm, and the relative chlorophyll content (SPAD) was noted using a chlorophyll meter (SPAD‐502). The fresh weight of the leaves was determined using an electronic balance accurate to 0.01 g. Leaves were then dried at 80°C for 48 hours until reaching a constant weight, at which point the dry weight was measured. The leaf dry matter content (LDMC) and specific leaf area (SLA) were calculated, representing the ratio of dry weight to fresh weight and the ratio of LA to dry weight, respectively.

The contents of flavonoids from one and two buds, including K‐3‐O‐*β*‐D‐gal, K‐3‐O‐*β*‐D‐glu, Q‐3‐O‐*β*‐D‐gal, and Q‐3‐O‐*β*‐D‐glu, were determined and extracted using high‐performance liquid chromatography (HPLC) following the methodology outlined by Liang, Qian, and Yao (2005).

### 2.2 Data Analysis

Phenotypic data underwent descriptive statistical analysis utilizing R software, version 3.6. Variance analysis for all traits was conducted employing a linear model, articulated as *X*_*ijk*_ = *μ* + *P*_*i*_ + *C*_*j*(*i*)_ + *ε*_ijk_, where *X* represents phenotypic individual observations, *μ* represents the population average, and *P*_*i*_ represents effect at place *i* (*i* = 1, 2, 3, 4, 5), *C*_*j*(*i*)_ represents the effect of the *j* clone in the *i* origin (*j* = 1, 2, …, 20), and *ε*_*ijk*_ represents residual.

The effects within and between origin clones, excluding the overall mean, were treated as random variables. ANOVA analysis was conducted using PROC GLM in SAS software (SAS Institute Inc. SAS/STAT software, v8) to investigate differences both between and within the origin clones. The variance components, namely, *σ*_*p*_^2^ (between the origins), *σ*_*c*(*p*)_^2^ (within the origins) and *σ*_*e*_^2^ (residual), were estimated based on the previously mentioned linear model. The coefficient of variation (CV) was calculated using the following formula: *CV* = *δ*_*p*_/*μ*, where *δ*_*p*_ represents the standard deviation of the phenotype and *μ* represents the mean value of the phenotype. The genetic correlation matrix and phenotypic correlation matrix between the two traits were calculated, and significance tests were conducted using the asreml software package.

### 2.3 RNA‐Seq

RNA extraction was conducted from one leaf and two buds of each hawk tea clone sample utilizing the RNA rapid extraction kit (Beijing, China). For quality control, each sample purity of OD260/280 was between 2.0 and 2.2 and RIN value of ≥ 8.0. Subsequently, equal amounts of total RNA from each sample were pooled, and the task of conducting transcriptome sequencing was entrusted to Hangzhou Kaitai Biotechnology Co. Ltd. In this process, the utmost accuracy in our transcriptome sequencing results was ensured by utilizing high‐quality transcript assembly, which combined second‐generation transcriptome sequencing with third‐generation full‐length transcriptome sequencing.

The raw image data generated from the second‐generation high‐throughput sequencing instrument, Illumina NovaSeq 6000, were subjected to base calling to convert them into sequence data, resulting in the acquisition of raw reads. It is important to note that these raw reads may potentially contain adapters or low‐quality base reads, which have the potential to adversely affect subsequent analyses. Therefore, it is imperative to perform data filtering to ensure the integrity of the information analysis process. In the context of quality control sequencing, the quality of the bases plays a critical role in achieving high sequencing accuracy (Li, Nordborg, and Li 2004). Q20 serves as a primary criterion for assessing data quality. Attainment of Q20 greater than 85% signifies that over 85% of the bases exhibit a sequencing accuracy rate of 99% (Baid et al. 2023). To achieve this, the data are disconnected from the sequencing platform, and a multistep data filtering process is subsequently executed, as detailed below:

- Reads with joint contamination greater than 5 bp were excluded from the dataset. In the case of double‐ended sequencing, both ends of the reads were discarded if one end exhibited splicing contamination.
- Reads with a quality score (*Q*) below 15, encompassing more than 30% of their length, were eliminated. In the context of double‐ended sequencing, if one end contained low‐quality reads, both ends were removed.
- Reads that contained more than 5% of the base “*N*” were filtered out. In the case of double‐ended sequencing, if one end contained more than 5% “*N*” bases, that specific end was excluded from the analysis.

Three generations of sequencing data were acquired utilizing Oxford Nanopore Technologies (ONT) sequencers. ONT sequencing boasts extended read lengths and high throughput, making it particularly advantageous in genome assembly, transcriptome assembly, epigenetic modification studies, and various other research domains (Zhang et al. 2023). The data filtering process was executed as follows: initial data assessment and statistics were performed using NanoPlot, followed by joint processing using Porechop. Subsequently, mass filtration was conducted with Nanofilt. Finally, NanoPlot was employed once more for comprehensive data statistics and evaluation of the resulting clean data. The merge assembly approach was employed to consolidate multiple samples into an initial transcriptome set. In cases where the sample size exceeded 20 samples, a random selection method was adopted, grouping them into sets of three, ensuring the inclusion of a total of 15 samples in the subsequent assembly process. The integration of NGS data and ONT data was accomplished using the default parameters of rnaspades v3.15.2, with the resulting transcripts fasta file serving as the foundation for subsequent analyses. To gauge the quality of assembly, reads were aligned to the assembled transcripts fasta using bowtie2 v2.4.2 to calculate the mapping rate, where a higher mapping rate is generally indicative of superior assembly integrity (Hyten et al. 2010). Assessment of transcript assembly integrity was carried out using BUSCO v5.0.0.

### 2.4 SNP Calling

STAR2.3 was employed for the comparison, and GATK4 was utilized for SNP calling. With *Litsea cubeba* as the reference, read mapping was conducted using STAR, information was appended to the BAM files using "Add or Replace Read Groups," and repeated reads were annotated using "Mark Duplicates." The BAM files were subsequently subjected to validation using "Validate SamFile," while splice reads underwent processing through "Split NCigar Reads." SNP calling was executed with "Haplotype Caller," and VCF merging was accomplished with "MergeVcfs." Variation filtration was applied using "Variant Filtration," and variants were extracted using "Select Variants," retaining only those reads that passed the filtration criteria. Mutation statistics were generated with Vcftools, and data visualization was performed using R packages.

Data conversion was carried out with vcf2phy to ensure that 90% of individuals possessed base information at the same site. Evolutionary trees were constructed using IQTrees, and for phylogenetic tree visualization, ggtree was employed. The approach constructed phylogenetic trees from allele frequency data by evaluating genetic distances among individuals within the population. Subsequently, data conversion was conducted using plink, followed by PCA analysis utilizing Smartpca, and the results were visualized through ggplot2. This approach utilized allele frequencies for genotype virtual variable transformation and PCA analysis to map individual‐level spatial sequencing relationships, facilitating the investigation of genetic structure and differentiation at the population level. Structural analysis was performed using admixture, with *K* values ranging from 2 to 5 chosen for display. This approach involved constructing a cluster model from multilocus genotype data, applying a mixed population model to depict genetic structure, calculating the *K* value to represent allelic variation frequency types, and determining the potential subpopulation count using the *K* value. Furthermore, Gmap was utilized to forecast the mapping of three generations of transcriptome sequences (CDS) onto the reference genome, determining their structural positions.

### 2.5 Transcriptome Association Analysis

Plink was employed for data transformation, and the association between SNP sites and flavonols was analyzed using a general linear model (GLM). The filtering criterion was set to –Log10(*p*) > 6.0. LD Block Show and Show LD SVG were utilized to construct LD blocks within the GWAS locus. The top 10 most significant loci were selected for each trait. The regions of interest extended 100 kb base pairs upstream and downstream of each significant association site, resulting in the analysis of a total of 200 kb base pair regions.

## 3 Results

### 3.1 Genetic Variation in DBH, Leaf Traits, and Flavonoid Content in Hawk Tea

The results of variance analysis (Table 1) indicate that, except for DBH, the origin significantly influenced leaf traits and flavonoid content (*p* < 0.001). The origin's impact accounted for 0.01% to 57.83% of the total variation in leaf traits and 0.57% to 31.69% of the total variation in flavonoid content. Among leaf traits, the clonal population in the KY area exhibited the highest values for LW, LA, LP, SPAD, and SLA, which were 4.57, 35.35, 31.51, 48.22, and 66.16, respectively (Table 2).

**TABLE 1:** Variance analysis and genetic parameter estimation of leaf traits, DBH, and flavonoid content.

Traits	Mean ± SD	CV	*σ*_*p*_^2^	*σ*_*c*(*p*)_^2^	*σ*_*e*_^2^
DBH	10.93 ± 1.83	31.64	13.07	62.44	61.75
LL	11.51 ± 1.29	15.55	41.59***	1.61	2.99
LW	3.53 ± 0.68	19.26	8.70***	0.32	0.45
LPL	1.15 ± 0.27	23.48	0.40***	0.06	0.07
LT	0.26 ± 0.89	342.31	0.14***	0.00	0.01
LA	25.11 ± 3.38	29.39	57.73***	12.17	54.97
LP	25.59 ± 0.83	18.87	48.52***	6.73	23.49
SPAD	45.80 ± 1.18	11.31	57.83***	21.31	24.75
LDMC	0.52 ± 0.04	7.69	0.01***	0.00	0.00
LS	3.34 ± 0.65	19.46	3.89***	0.27	0.35
SLA	48.7 ± 1.97	10.20	33.32***	65.51	223.94
K‐3‐O‐*β*‐D‐gal	0.64 ± 0.45	70.31	1.40***	0.15	0.18
K‐3‐O‐*β*‐D‐glu	2.39 ± 1.75	73.22	23.31***	2.20	2.56
Q‐3‐O‐*β*‐D‐gal	0.48 ± 0.30	62.50	0.57***	0.07	0.08
Q‐3‐O‐*β*‐D‐glu	8.88 ± 0.20	69.82	31.69***	25.71	28.93

Abbreviations: CV, coefficient of variation; DBH, diameter at breast height (cm); K‐3‐O‐*β*‐D‐gal (mg/g dry weight); K‐3‐O‐*β*‐D‐glu (mg/g dry weight); LA, leaf area (cm^2^); LDMC, leaf dry matter content; LL, leaf length (cm); LW, leaf width (cm); LP, leaf perimeter (cm); LPL, leaf petiole length (cm); LS, leaf shape index; LT, leaf thickness (cm); Q‐3‐O‐*β*‐D‐gal (mg/g dry weight); Q‐3‐O‐*β*‐D‐glu (mg/g dry weight); SLA, specific leaf area; SPAD, the relative chlorophyll content; *σ*_*c*(*p*)_^2^, variation within the origin; *σ*_*e*_^2^, residual*σ*_*p*_^2^, variation between the origin.

*p < 0.05, **p < 0.01, ***p < 0.001.

**TABLE 2:** Average leaf traits, DBH, and flavonoids in five areas.

	DZ	KY	MT	ZA	XS
DBH	11.03 ± 1.71	10.66 ± 1.03	13.05 ± 1.42	9.68 ± 1.87	10.25 ± 0.98
LL	10.65 ± 1.35c	12.48 ± 1.23b	10.61 ± 1.02c	10.25 ± 1.42c	13.57 ± 1.28a
LW	3.21 ± 0.37c	4.57 ± 0.39a	3.50 ± 0.38b	2.79 ± 0.29d	3.59 ± 0.27b
LPL	1.13 ± 0.20b	1.20 ± 0.33ab	1.33 ± 0.25a	0.94 ± 0.18c	1.15 ± 0.23ab
LT	0.21 ± 0.04b	0.22 ± 0.03b	0.24 ± 0.03b	0.41 ± 0.08a	0.21 ± 0.03b
LA	20.84 ± 2.98c	35.35 ± 2.91a	22.07 ± 2.52c	17.52 ± 2.84d	29.76 ± 1.66b
LP	23.00 ± 2.76c	31.51 ± 2.78a	22.98 ± 1.68c	21.05 ± 2.70c	29.40 ± 2.87b
SPAD	46.84 ± 1.52a	48.22 ± 2.80a	47.69 ± 0.36a	41.29 ± 5.45b	44.94 ± 3.60a
LDMC	0.54 ± 0.03a	0.54 ± 0.02a	0.50 ± 0.04bc	0.52 ± 0.04b	0.49 ± 0.03c
LS	3.37 ± 0.63b	2.75 ± 0.33c	3.08 ± 0.51b	3.72 ± 0.66a	3.79 ± 0.39a
SLA	38.95 ± 1.42d	66.16 ± 0.70a	44.15 ± 0.91c	33.53 ± 0.39e	60.87 ± 0.34b
K‐3‐O‐*β*‐D‐gal	0.21 ± 0.11b	0.66 ± 0.27a	0.91 ± 0.47a	0.62 ± 0.34a	0.79 ± 0.57a
K‐3‐O‐*β*‐D‐glu	0.70 ± 0.39c	2.54 ± 0.87ab	3.16 ± 1.43ab	2.12 ± 1.14b	3.44 ± 2.60a
Q‐3‐O‐*β*‐D‐gal	0.18 ± 0.06b	0.53 ± 0.16a	0.58 ± 0.33a	0.49 ± 0.34a	0.60 ± 0.32a
Q‐3‐O‐*β*‐D‐glu	2.36 ± 1.18c	9.61 ± 3.06b	9.82 ± 2.38b	8.77 ± 2.15b	13.81 ± 1.89a

*Note:* Values with different superscripts in the same column significantly differ at the 0.05 level.

Abbreviations: DBH, diameter at breast height (cm); K‐3‐O‐*β*‐D‐gal (mg/g dry weight); K‐3‐O‐*β*‐D‐glu (mg/g dry weight); LA, leaf area (cm^2^); LDMC, leaf dry matter content; LL, leaf length (cm); LP, leaf perimeter (cm); LPL, leaf petiole length (cm); LS, leaf shape index; LT, leaf thickness (cm); LW, leaf width (cm); Q‐3‐O‐*β*‐D‐gal (mg/g dry weight); Q‐3‐O‐*β*‐D‐glu (mg/g dry weight). SLA, specific leaf area; SPAD, the relative chlorophyll content.

Conversely, the LL, LW, LPL, LA, LP, and SPAD of clonal populations in the ZA area were the smallest, measuring 10.25, 2.79, 0.94, 17.52, 21.05, and 41.29, respectively. The maximum LL observed in the clonal population was 13.57 in the XS area (Table 2).

Regarding flavonoid content, Q‐3‐O‐*β*‐D‐glu, K‐3‐O‐*β*‐D‐gal, and K‐3‐O‐*β*‐D‐glu exhibited the highest values in clonal populations from the XS area, measuring 3.44, 0.60, and 13.81, respectively. Conversely, the contents of these four flavonoids in the clonal population from the DZ area were the smallest, measuring 0.21, 0.70, 0.18, and 2.36, respectively.

For DBH, the clonal variation between and within origins did not reach a significant level (*p* > 0.05). In contrast, for leaf traits and flavonoid content, the primary source of genetic variation stemmed from the variation between populations.

### 3.2 Correlations Among DBH, Leaf Traits, and Four Types of Flavonoids

**FIGURE 2:**
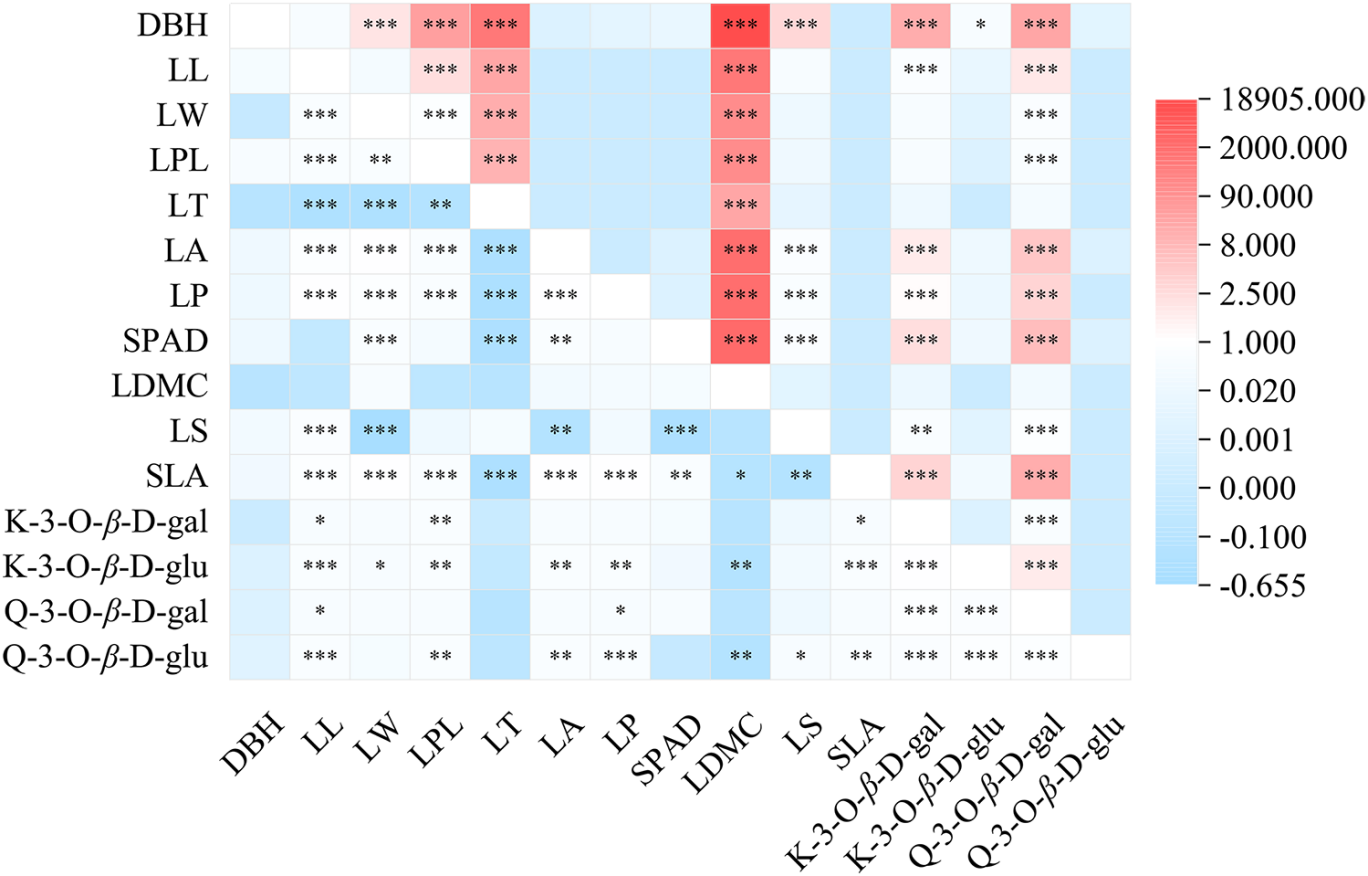
Genetic correlation (above) and phenotypic correlation (below) among DBH, leaf traits, and four flavonoids. DBH, diameter at breast height (cm); LL, leaf length (cm); LW, leaf width (cm); LA, leaf area (cm^2^); LT, leaf thickness (cm); LP, leaf perimeter (cm); LS, leaf shape index; LPL, leaf petiole length (cm); SPAD, the relative chlorophyll content; LDMC, leaf dry matter content; SLA, specific leaf area; K‐3‐O‐*β*‐D‐gal (mg/g dry weight); K‐3‐O‐*β*‐D‐glu (mg/g dry weight); Q‐3‐O‐*β*‐D‐gal (mg/g dry weight); and Q‐3‐O‐*β*‐D‐glu (mg/g dry weight). *p < 0.05, **p < 0.01, ***p < 0.001.

The phenotypic and genetic correlations among DBH, leaf traits, and the four flavonoids are presented in Figure 2. The results indicated that the phenotypic and genetic correlation coefficients among K‐3‐O‐*β*‐D‐glu, DBH, and leaf traits were not statistically significant, with coefficients ranging from 0.2064 to 0.4086.

On the other hand, Q‐3‐O‐*β*‐D‐gal and K‐3‐O‐*β*‐D‐gal exhibited significant positive correlations with LL, LA, LP, SPAD, LS, and SLA. Additionally, K‐3‐O‐*β*‐D‐gal was positively correlated with LW, and LPL showed a significant positive correlation. Furthermore, Q‐3‐O‐*β*‐D‐gal, Q‐3‐O‐*β*‐D‐glu, and K‐3‐O‐*β*‐D‐gal displayed significant positive correlations with DBH, suggesting that DBH can serve as an indirect selection indicator for hawk tea flavonoids.

Regarding the correlations between the four flavonoid contents, Q‐3‐O‐*β*‐D‐gal and Q‐3‐O‐*β*‐D‐glu (*p* < 0.01), Q‐3‐O‐*β*‐D‐gal and K‐3‐O‐*β*‐D‐gal, Q‐3‐O‐*β*‐D‐glu and K‐3‐O‐*β*‐D‐gal (*p* < 0.001) exhibited statistically significant phenotypic correlations (Figure 2). Additionally, significant genetic correlations were observed between Q‐3‐O‐*β*‐D‐gal and K‐3‐O‐*β*‐D‐gal, as well as between Q‐3‐O‐*β*‐D‐glu and K‐3‐O‐*β*‐D‐gal (*p* < 0.001).

### 3.3 Second‐ and Third‐Generation Sequencing and SNP Statistics

**FIGURE 3:**
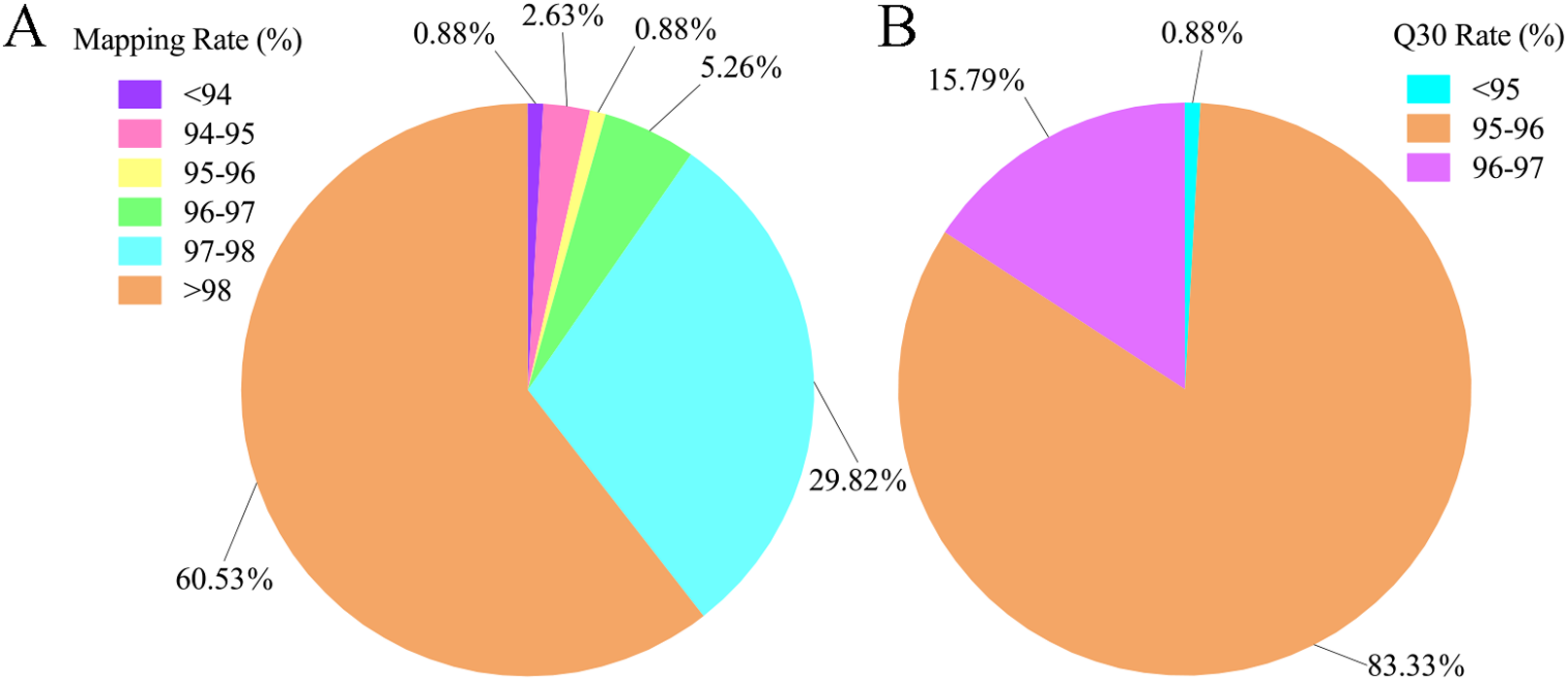
Statistics of sequencing results. (A) The distribution of alignment rates. (B) The distribution of Q30 rates.

Based on the Clean Data statistics for each hawk tea sample, the data utilization rate falls within the range of 93.15% to 98.81%. The distribution of GC content ranges from 46.37% to 49.36%. With the exception of KY13, all other samples exhibited mapping values exceeding 94% (Figure 3A), and the transcript integrity was notably high at 96.3%, as assessed by BUSCO software. In general, assembled results typically fall within the range of 70% to 98% and are deemed suitable for subsequent analysis (Kishi‐Kaboshi et al. 2022). Furthermore, more than 94% of the bases exhibit a Q30 quality score (Figure 3B). These observations collectively indicate that the sequencing data possess high quality and are suitable for sequence fragment assembly and subsequent analysis. The raw reads have been deposited in NCBI and are accessible under BioProject PRJNA992466.

**FIGURE 4:**
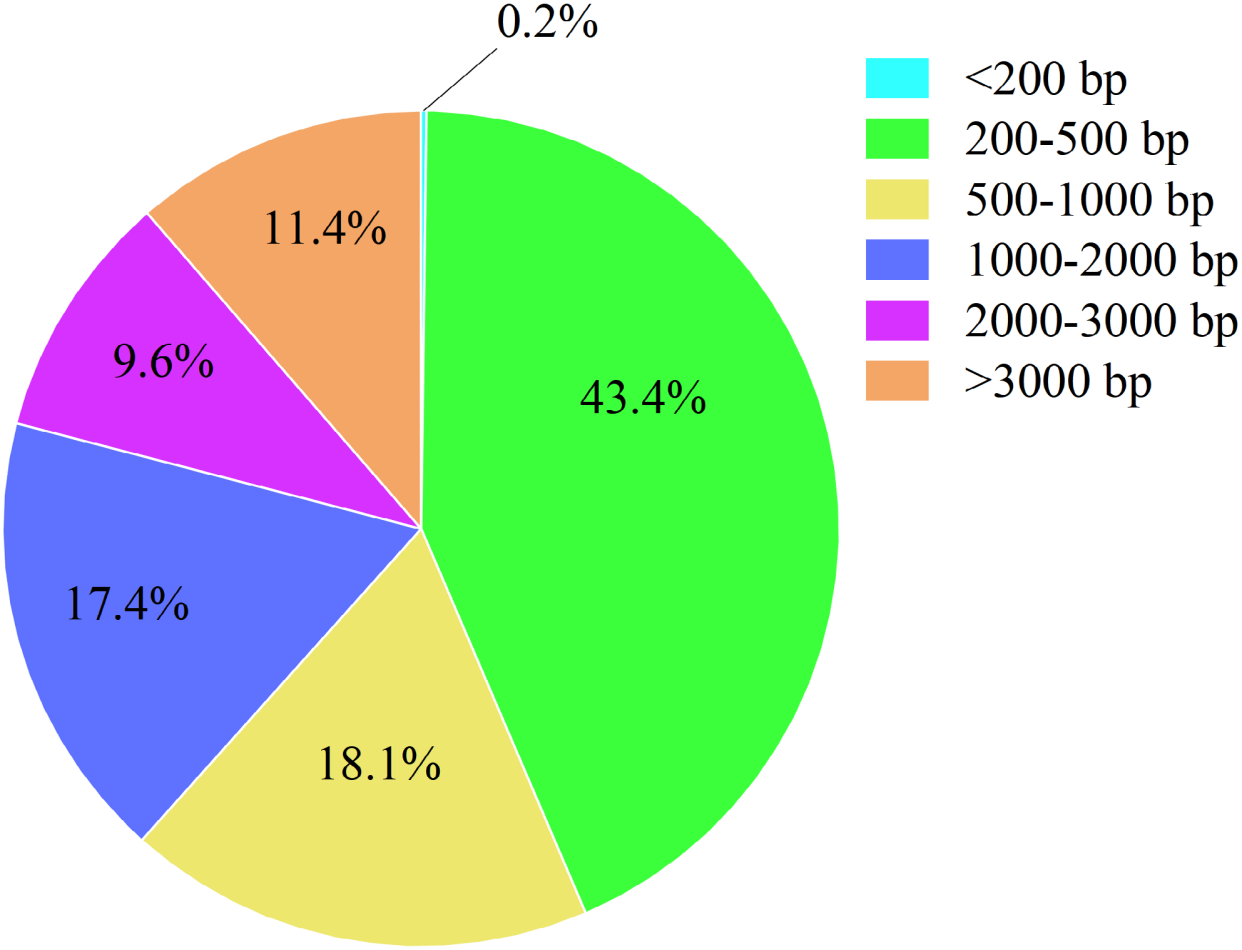
Statistical distribution of transcription length sequence.

Following the assembly and splicing process, a total of 349,993 transcripts were obtained, comprising 449,816,814 bases. The average transcript length was 1285 bp, with an N50 length of 2494 bp. Notably, transcripts falling within the 200–500 bp range constituted a relatively substantial portion, accounting for 43.4% of the total transcripts (Figure 4).

**FIGURE 5:**
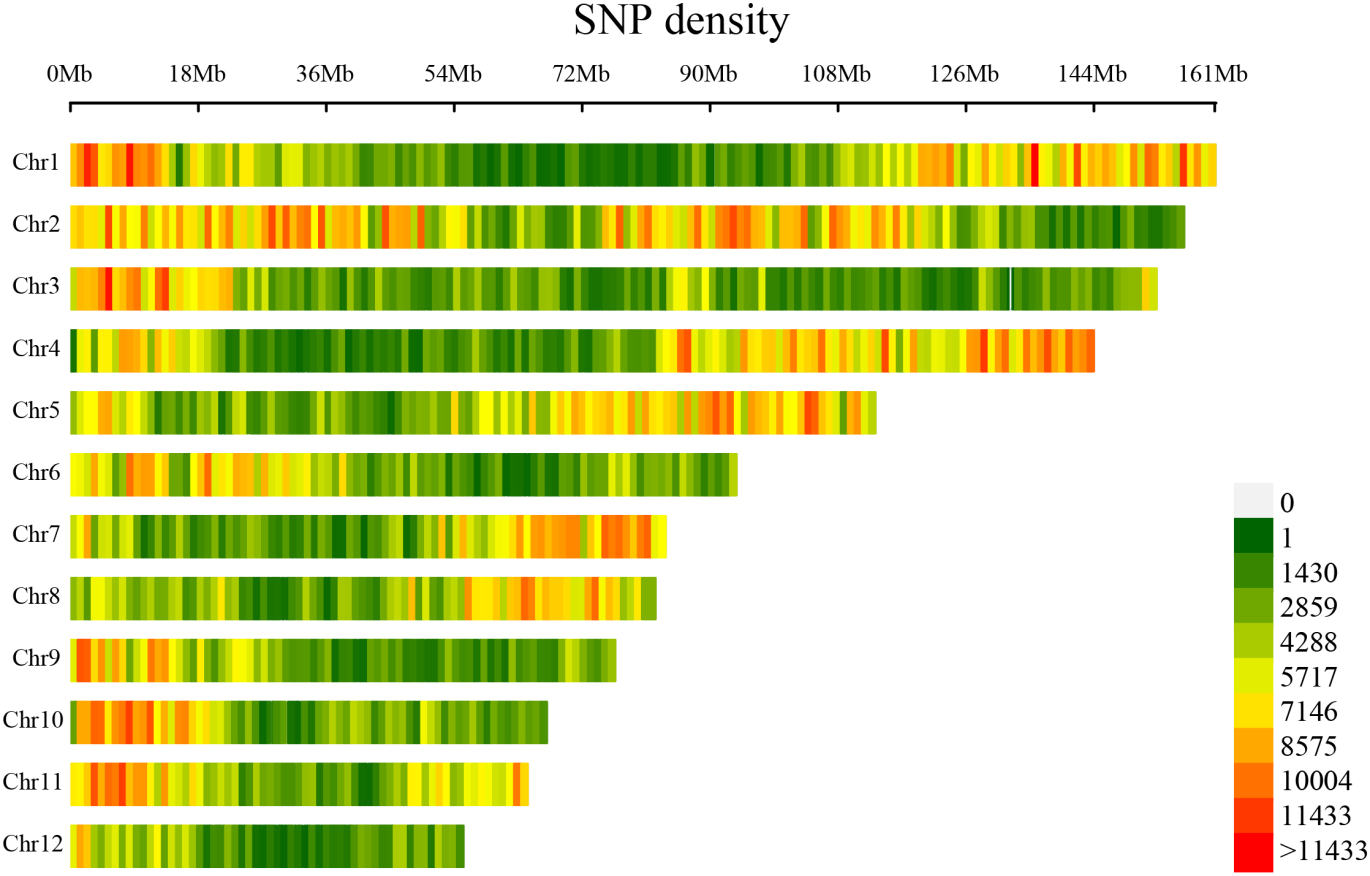
Distribution of SNP density across chromosomes. (Different colored regions indicate varying SNP counts across chromosomes.)

The valid data obtained were compared with the *Litsea* genome, yielding an average alignment rate of 85.37%, falling within a confidence interval of 72.53%—88.59% (Figure 3A). Subsequently, SNP calling was conducted using GATK, resulting in each sample containing more than 600,000 SNPs (Figure 5). Notably, the Phred values for the majority of these sites exceeded 1000. Figure 5 illustrates the distribution of these SNPs across chromosomes, revealing that, apart from chromosome 12, each of the other chromosomes harbored more than 10,000 SNPs.

### 3.4 Genetic Evolutionary Analysis

**FIGURE 6:**
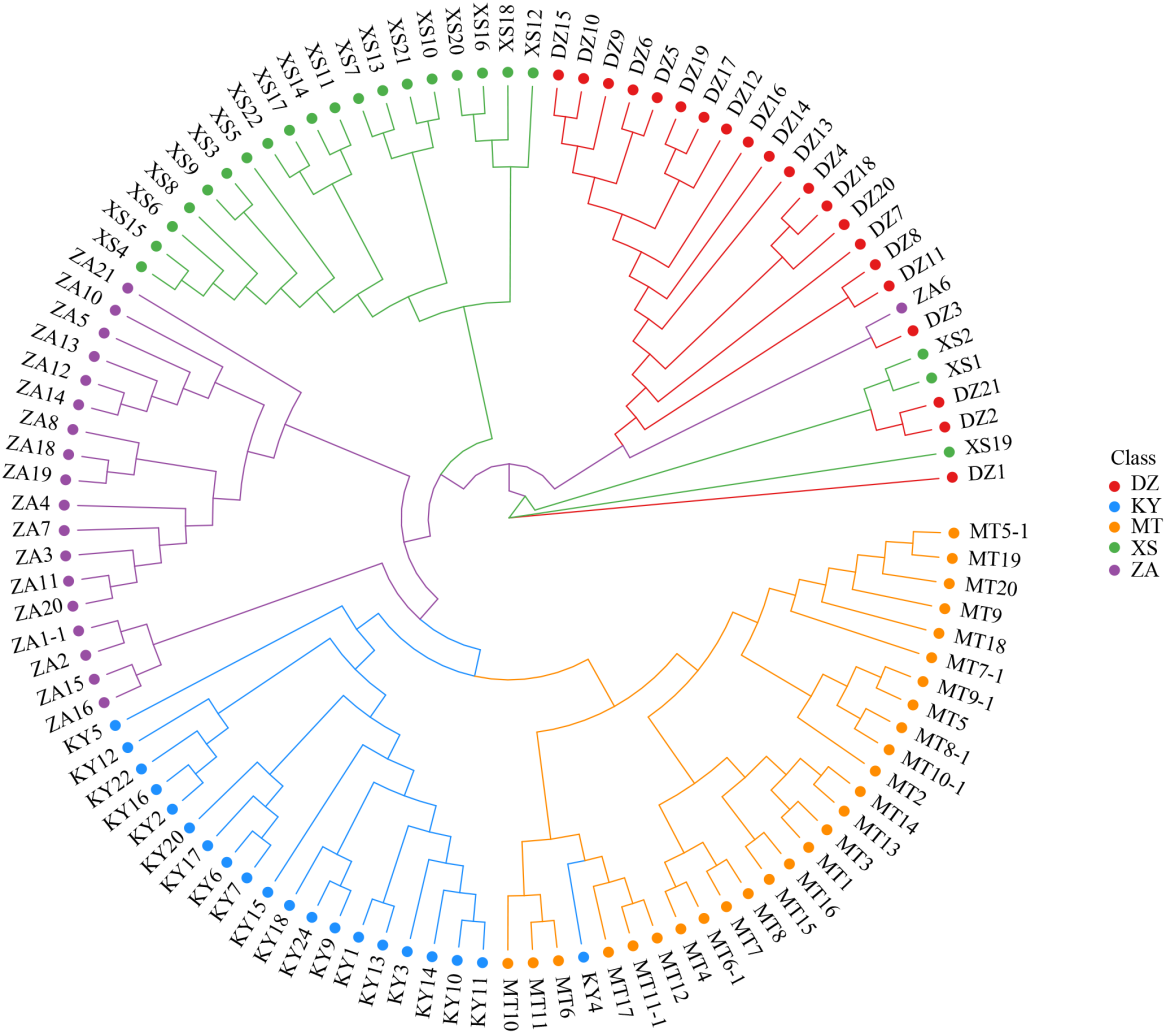
Phylogenetic tree of hawk tea populations constructed based on genetic distance. (Red represents DZ area, blue represents KY area, orange represents MT area, green represents XS area, and purple represents ZA area.)

Based on the phylogenetic tree constructed using the neighbor‐joining clustering method, which was based on genetic distance, the results (Figure 6) revealed the division of 109 hawk tea clones from five different regions into five distinct subgroups. The first subgroup primarily consisted of clones from DZ, XS, and ZA, while the second subgroup was predominantly composed of clones from XS. Clones from the ZA provenance dominated the third subgroup, whereas the fourth subgroup was mainly comprised of clones from KY. The fifth subgroup predominantly consisted of clones from MT and KY.

**FIGURE 7:**
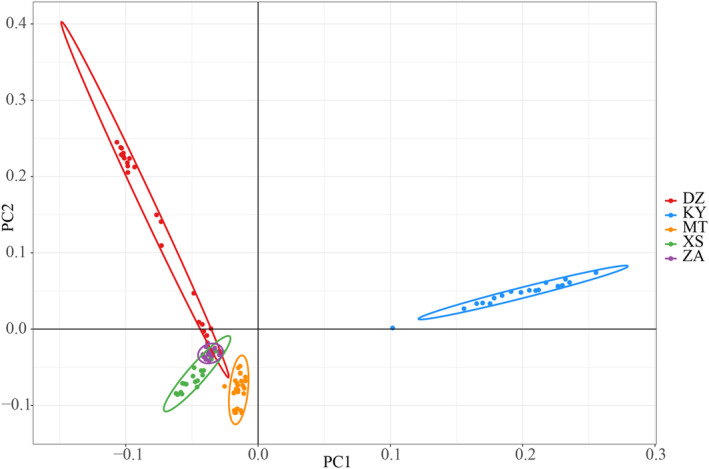
Principal component analysis of hawk tea. (Red represents DZ area, blue represents KY area, orange represents MT area, green represents XS area, and purple represents ZA area.)

**FIGURE 8:**
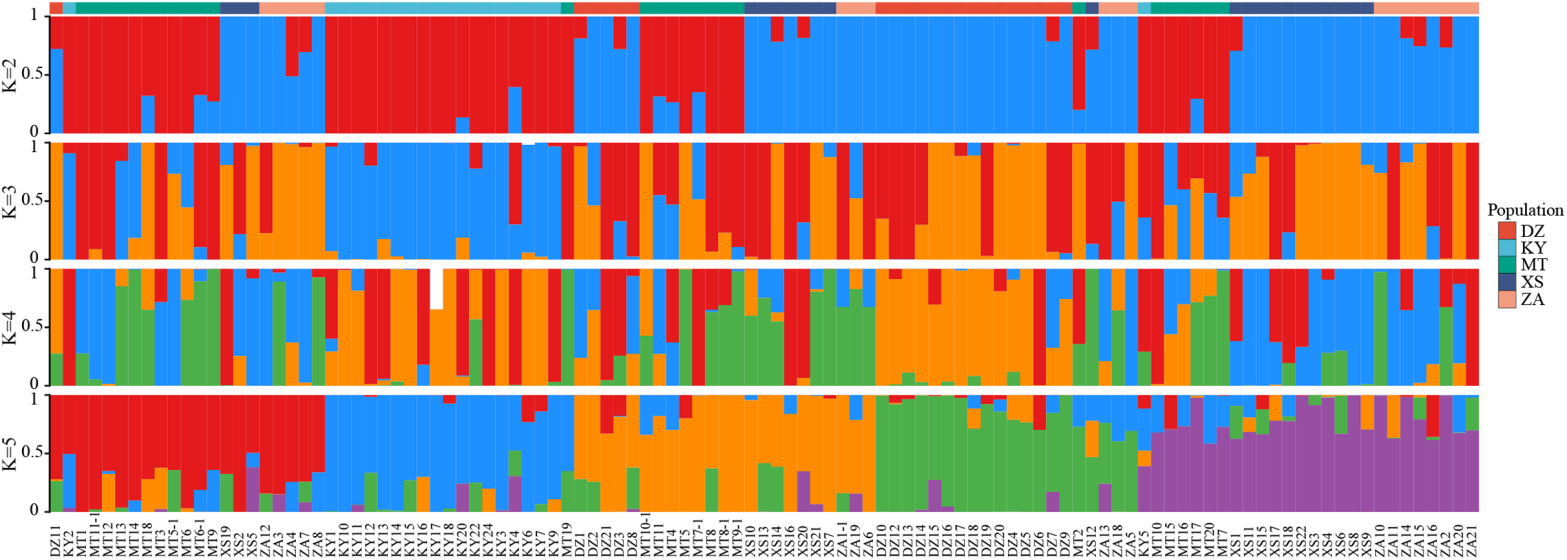
Results of the Bayesian clustering analysis conducted using STRUCTURE. (Highlighting the clustering patterns of genetic components across 2–5 groupings.)

Further insights into the clustering patterns of all samples were obtained through PCA analysis of the transformed data (Figure 7). This analysis categorized the samples into five distinct groups, with DZ, KY, MT, and XS forming four separate categories, while ZA clustered together with DZ and XS. To delve into the population structure of the studied materials, Admixture software was employed (Figure 8). The results indicated that when *K* = 5, the 109 hawk tea clones were classified into five subgroups, with the lowest cross‐verification error rate observed at this value.

Inadequate control for population structure may lead to false‐positive results in association analyses (Joo et al. 2015). The population was stratified into five subgroups through the neighbor‐joining clustering method based on genetic distance (Figure 6), the principal component analysis (Figure 7), and application of admixture software (Figure 8). Results of these three methods showed that the hawk tea had differentiation at the population level, and there was genetic difference in geographical distribution patterns at the subgroup level, which could be used for the next association analysis.

### 3.5 Transcriptome‐Wide Association Analysis of Flavonoids

The primary content of the four flavonoids in the tender shoots of hawk tea has been determined, and significant variations in flavonoid content among different cultivation regions and clones of hawk tea have been observed (Table 1). After filters were applied based on criteria such as marker missing rate, sample missing rate, and minor allele frequency (MAF), a total of 235 high‐quality SNPs associated with flavonoids were identified, of which 84 demonstrated statistical significance. Among these SNPs, 66 (78.57%) were found to be situated in intergenic regions. Further breakdown reveals that 10 were located in upstream regions, 23 in introns, 9 in downstream regions, 15 were represented in missense variants, and 9 were synonymous variants. Moreover, functional annotations were available for 44 of these SNPs (Table S1). In hawk tea's tender shoots, significant SNPs associated with the four flavonoids were identified, with totals of 11, 7, 30, and 36 for each respective flavonoid. It is important to mention that only a limited number of SNPs were localized within gene regions (Table 3).

**TABLE 3:** Summary of the significant SNPs by associated analysis.

Traits	Significant SNPs	SNPs in genic region	Associated genes	Chromosome	SNP position	–Log10 *p*	Annotation	KEGG pathways	KO
K‐3‐O‐*β*‐D‐gal	11	8	3	CM022944.1	121937053	7.1918	Cytochrome P450 CYP4/CYP19/CYP26 subfamilies	CYP86B1; fatty acid omega‐hydroxylase	K09590
				CM022946.1	13199858	6.0132	Selenium‐binding protein	SELENBP1; methanethiol oxidase	K17285
				CM022952.1	25558941	6.7258	UDP‐glucuronosyl and UDP‐glucosyl transferase	UGT74B1; N‐hydroxythioamide S‐beta‐glucosyltransferase	K11820
K‐3‐O‐*β*‐D‐glu	7	4	1	CM022944.1	121937053	6.1803	Cytochrome P450 CYP4/CYP19/CYP26 subfamilies	CYP86B1; fatty acid omega‐hydroxylase	K09590
Q‐3‐O‐*β*‐D‐gal	30	12	3	CM022945.1	39681060	7.0556	Predicted importin 9	IPO9, RANBP9; importin‐9	K20224
				CM022945.1	28136888	6.3667	Serine/threonine protein phosphatase 2A, regulatory subunit	PPP2R5; serine/threonineprotein phosphatase 2A regulatory subunit B′	K11584
				CM022947.1	7598503	6.3111	Dihydrolipoamide acetyltransferase	DLAT, aceF, pdhC; pyruvate dehydrogenase E2 component (dihydrolipoyllysine residue acetyltransferase)	K00627
K‐3‐O‐*β*‐D‐glu	36	20	6	CM022953.1	47477583	7.1035	Scaffold‐/matrix‐specific factor hnRNP‐U/SAF‐A contains SPRY domain	DLD,1pd,pdhD; dihydrolipoy1 dehydrogenase	K00382
				CM022947.1	2558160	7.0773	—	ppc; phosphoenolpyruvate carboxylase	K01595
				CM022946.1	12277330	6.8386	Sterol O‐acyltransferase/ diacylglycerol O‐acyltransferase	P4HA; prolyl4‐hydroxylase	K00472
				CM022947.1	7529974	6.7878	—	DGAT1; diacylglycerol O‐acyltransferase 1	K11155
				CM022950.1	79,888,055	6.0975	Mitogen‐activated protein kinase	LEU1; 3‐isopropylmalate dehydratase	K01702
				CM022944.1	12,714,282	6.0191	—	HPR2‐3; glyoxylate/hydroxypyruvate reductase	K15919

**FIGURE 9:**
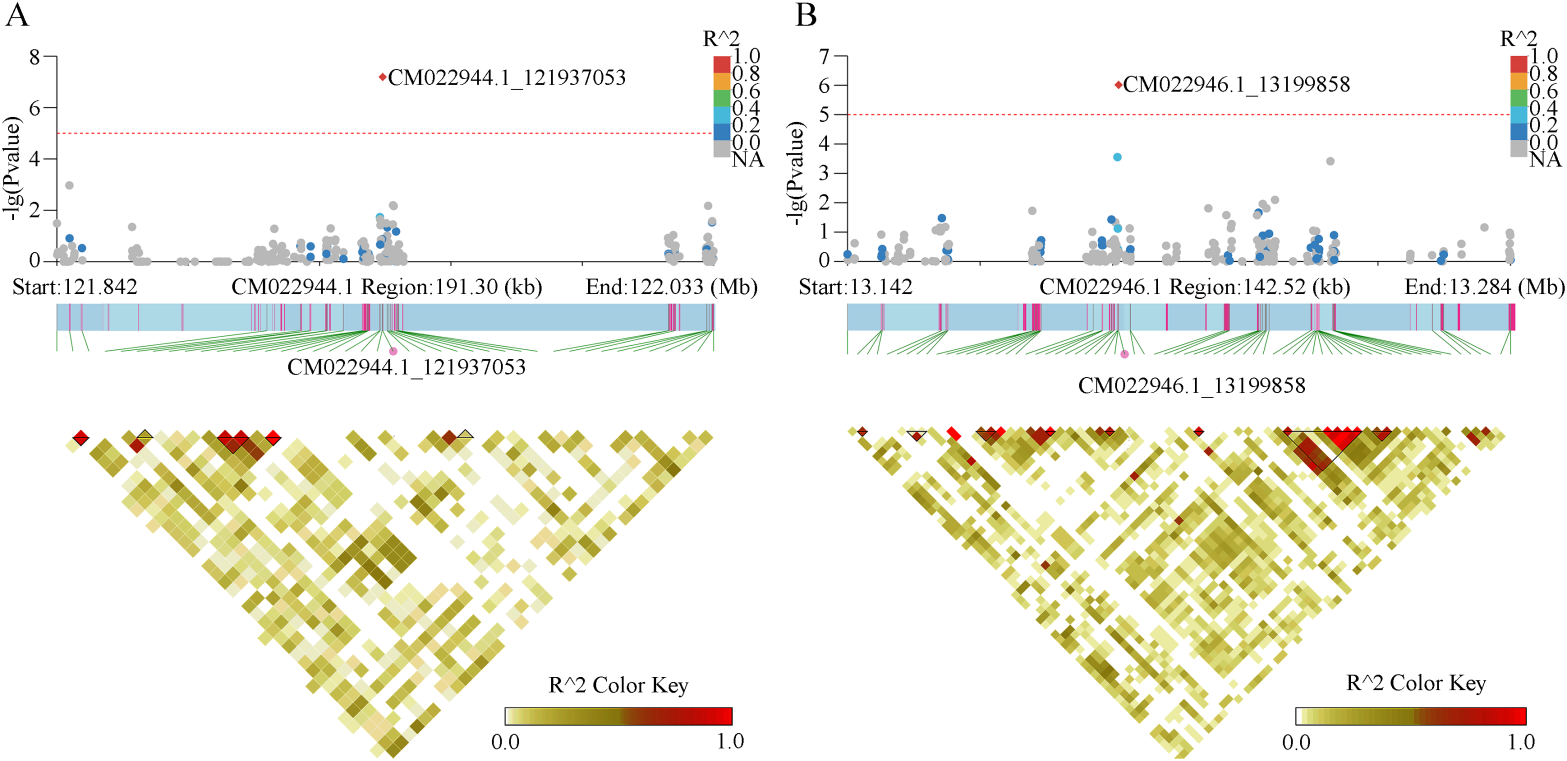
Association screening for the SNP locus grounded on kaempferol‐3‐O‐*β*‐D‐galactoside content. (A) Location of SNP locus 1,219,377,053 on the chromosome CM022944.1. (B) Location of SNP locus 13,199,858 on the chromosome CM022946.1.

Given the unavailability of the hawk tea genome, our investigation was constrained to genes exhibiting significant SNPs. A total of 44 protein‐coding genes presenting *p* values below 0.0001 were discerned (Table S1). Three genes, associated with K‐3‐O‐*β*‐D‐gal content, were categorized into three distinct functional classes: the cytochrome P450 subfamilies CYP4/CYP19/CYP26, selenoproteins, and uridine diphosphate glucose transferases (Figure 9). Regarding K‐3‐O‐*β*‐D‐glu, a solitary gene from the cytochrome P450 subfamilies CYP4/CYP19/CYP26 was identified. In contrast, Q‐3‐O‐*β*‐D‐gal content was linked to three genes, inclusive of those coding for dihydroceramide transferases. Moreover, six genes correlated with K‐3‐O‐*β*‐D‐glu content were also pinpointed (Table 3).

**FIGURE 10:**
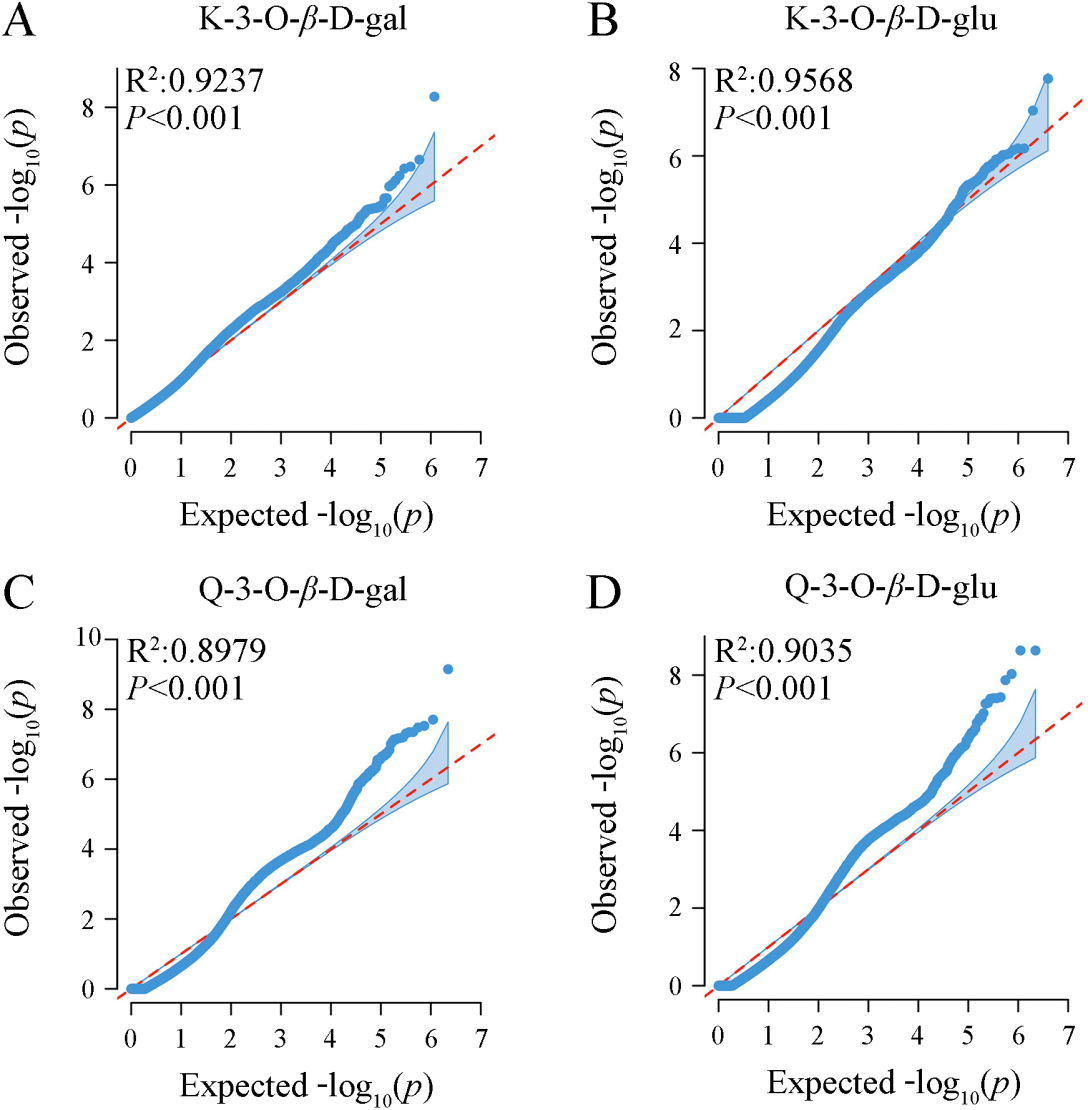
QQ map of *p* value distribution of SNP associated with flavonol‐related traits of hawk tea.

**FIGURE 11:**
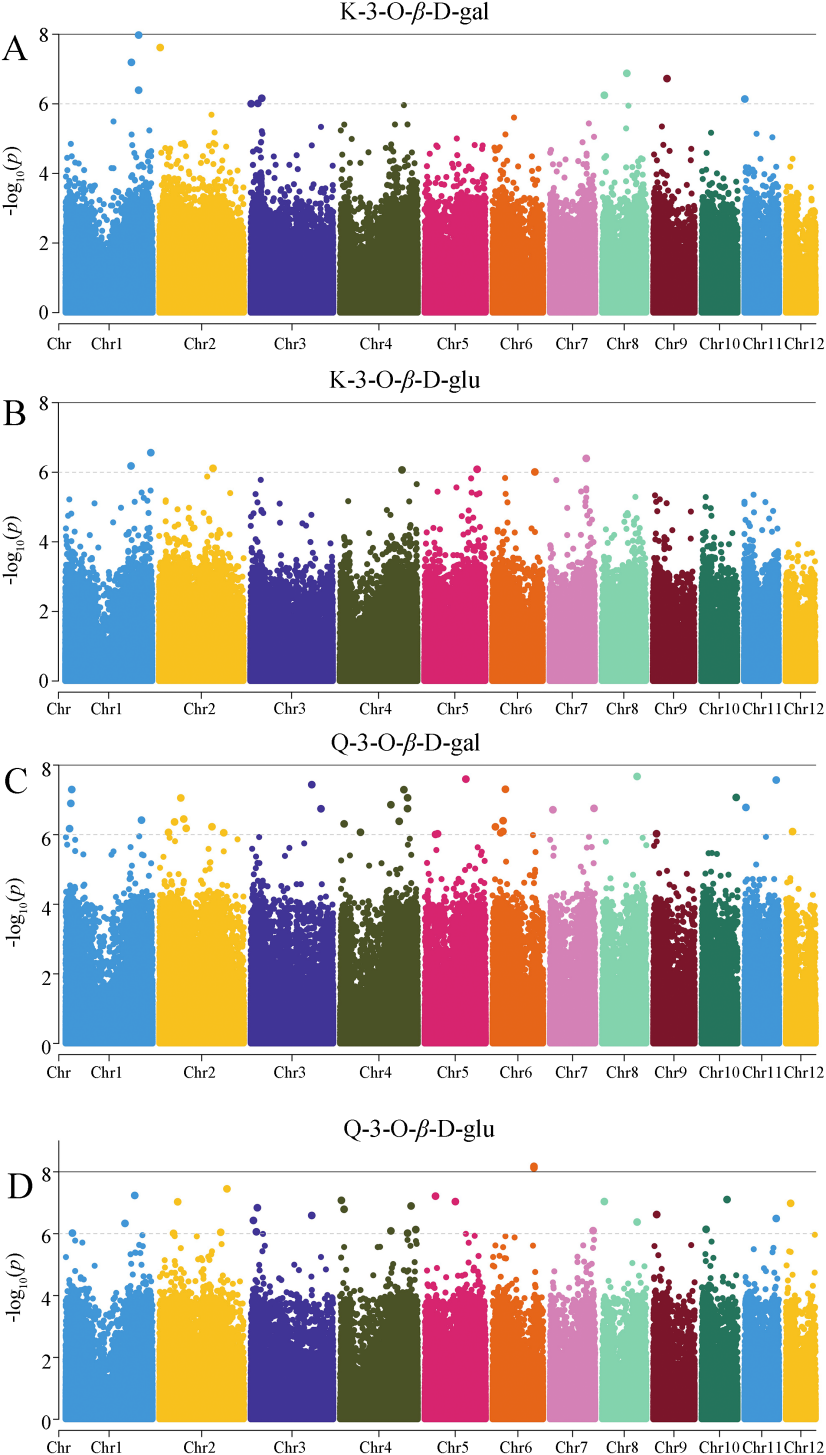
Manhattan plot of transcriptome‐wide association analysis for flavonoid‐related traits in hawk tea. The Bonferroni‐adjusted suggestive and significant thresholds are illustrated by black and gray dotted horizontal lines (−*log*10[*p*] values of 8 and 6, respectively). The *X* axis displays the chromosome numbers.

The examination of *p* value distributions from GLM association analyses for flavonol traits, K‐3‐O‐*β*‐D‐gal, K‐3‐O‐*β*‐D‐glu, Q‐3‐O‐*β*‐D‐gal, and Q‐3‐O‐*β*‐D‐glu (Figure 10) results showed that the loci with relatively high significance are situated in the upper right corner and are potential candidate loci associated with the flavonol trait. These points are located above the diagonal line, suggesting that the observed *p* values of the loci are higher than the expected values, which implies that the effects of these points exceed the random effects and further indicates that these loci are significantly related to the flavonol trait. Manhattan plots illustrating the *p* values from the association analyses for these four flavonoid traits are presented in Figure 11.

Within the SNP sites linked to K‐3‐O‐*β*‐D‐gal, 34 were identified, with 11 showing significant associations (Figure 11A). The polymorphism of SNPs primarily arises from transition (C‐T and G‐A) and transversion (C‐A, C‐G, G‐T, and A‐T) mutations. Among these sites, transitions constitute 55.88% and transversions make up 44.12% (Table S1). For K‐3‐O‐*β*‐D‐glu, 22 SNP sites were found, 7 of which were significantly associated (Figure 11B). In this context, transitions represent 27.27%, whereas transversions account for 72.73% (Table S1). Regarding Q‐3‐O‐*β*‐D‐gal, 104 SNP sites were identified, with 36 being significantly associated (Figure 11C). Here, transition mutations comprise 86.54%, and transversion mutations 13.46% (Table S1). Lastly, for Q‐3‐O‐*β*‐D‐glu, 75 SNP sites were noted, with 30 showing significant associations (Figure 11D). Among these, transition mutation sites are 69.33% and transversion mutation sites are 30.67% (Table S1).

## 4 Discussion

### 4.1 Genetic Variation of DBH, Leaf Traits, and Flavonoid Content of Hawk Tea

Guizhou Province, situated in southwest China, is distinguished by its extensive distribution of carbonate rocks and karst landforms (Zhang et al. 2022). This region stands out globally due to its intricate geographical features that cultivate a variety of microclimates, potentially leading to variations in plant characteristics and the concentration of active compounds (Xiong et al. 2023). Our research revealed that the differences in DBH across and within Guizhou regions were not statistically significant (Table 2), suggesting uniform growth patterns for hawk tea across the province. The diversity in leaf traits and flavonoid content primarily stemmed from the distinct habitats, highlighting that hawk tea's growth and development exhibit variation in response to the unique microclimatic conditions prevalent in Guizhou. This observation aligns with the findings of Hsiung et al. (2017), who noted that minor geoclimatic shifts can induce morphological and anatomical adaptations in leaves, facilitating plant survival and establishment in novel environments. This has profound implications for our understanding of plant survival, adaptation, and evolution. Factors such as temperature, sunlight intensity, and rainfall not only serve as fundamental prerequisites for plant growth but also significantly influence the composition of plant active components (Yu et al. 2015). Consequently, variations in the microclimate of different areas may also reflect in the regional differences in flavonoid content.

### 4.2 Correlation Among DBH, Leaf Traits, and Four Kinds of Flavonoids

The correlation coefficient serves as a crucial statistical tool for quantifying the relationship between two variables (Baak et al. 2020). In our analysis, significant positive correlations were observed between both Q‐3‐O‐*β*‐D‐gal and K‐3‐O‐*β*‐D‐gal with LL, LA, LP, SPAD values, LS, and SLA. Moreover, K‐3‐O‐*β*‐D‐gal also showed a significant positive correlation with LW and LPL (Figure 2). Given that the flavonol content influences the taste of hawk tea, our findings suggest that leaves with superior quality are more desirable for processing hawk tea. The significant positive correlation of Q‐3‐O‐*β*‐D‐gal, Q‐3‐O‐*β*‐D‐glu, and K‐3‐O‐*β*‐D‐gal with DBH (Figure 2) implies that DBH could serve as an indirect selection criterion for hawk tea content, hinting at a link between flavonol accumulation and tree age (Wang et al. 2022). The interrelations among the four flavonol contents indicate that their accumulation in hawk tea is contingent upon the planting environment and genetic factors. The genetic background determines the capacity of plants to adapt to environmental conditions. Differences in metabolite production have been observed between samples of the same species grown under varying environmental conditions (Table 2). Specific environmental factors have been identified as major sources of variation in intraspecies metabolism (Rubert‐Nason et al. 2023). For instance, abiotic factors such as soil nutrients and water availability can induce significant differences in the amount of compounds accumulated by plants in different regions (Liang, Qian, and Yao 2005). Plant traits emerge from the prolonged interplay between genetic attributes and environmental conditions (Florez et al. 2009). Optimal temperatures and altitudes can foster enhanced flavonol growth (Marotti et al. 2020). The presence of genetic traits within and among plant populations could facilitate a quicker adaptation to environmental shifts, allowing plants to survive, adapt, and evolve in new settings and consequently produce various flavonol classes (Agostini‐Costa 2022).

### 4.3 Second‐ and Third‐Generation Sequencing Data and SNP Statistics

Both second‐ and third‐generation transcriptome sequencing techniques were utilized. By employing the "three + two" model, the third‐generation full‐length transcriptome data were refined with the help of parameter‐free assembly data from the second generation, leading to the acquisition of high‐quality transcripts. The proportion of Q30 bases exceeded 94% (Figure 3B), underscoring the high quality of the sequencing data. Moreover, the mapping rates for the sequencing samples were predominantly above 94% (with the exception of KY13), signifying excellent data fidelity. The completeness of the transcripts, as assessed by BUSCO, reached an impressive 96.3%. The data generated were then aligned with the genome of *Litsea cubeba*, a species closely related, achieving an average mapping rate of 85.37% and identifying over 600,000 SNPs per sample (Figures 4 and 5). In conclusion, the "three + two" model implemented has proven to be an effective strategy for generating high‐quality transcripts for further analysis in this study.

### 4.4 Analysis of Population Genetic Structure of Hawk Tea

The determinants of association analysis outcomes are primarily governed by factors such as the quantity of SNPs, the diversity and scale of population materials, and the choice of statistical techniques (Kim et al. 2022). A notable challenge in association analysis is the potential for population structure to spuriously link target traits with unrelated genes, elevating the rate of false positives (Iwata et al. 2007). The efficacy of association analysis is maximized in populations with simple structures, where the likelihood of erroneous links is minimized (Kaler et al. 2020). Conversely, intricate population structures amplify linkage disequilibrium across the population, increasing the incidence of false associations between traits and gene polymorphisms (Iwata et al. 2007). Implementing population structure analyses can mitigate the rate of false associations, with strategies such as structural association analysis, principal component analysis, genomic control, and multidimensional scaling addressing the impact of population structure on association studies (Hu and Ziv 2008). Three methodologies were employed to examine the genetic structure of hawk tea populations (Figures 6, 7, 8). The outcomes from these three methodologies were consistent, classifying 109 clones into five subgroups, thereby enabling their correlation with quantitative traits.

### 4.5 Association Analysis of Flavonols

The combined analysis of expression profiles, metabolic profiles, and transcriptome association studies stands as a crucial approach for investigating quantitative traits within complex metabolic systems (Robinson et al. 2007). In the case of hawk tea, flavonols represent the primary constituents. Nonetheless, the intricate nature and extensive labor required for qualitative and quantitative assessments have limited research into the SNP sites associated with anabolic metabolism and its genetic underpinnings (Ye et al. 2021). Metabolic data, transcriptome expression profiles, and high‐density variant findings derived from "three + two" mode sequencing were leveraged in conducting a quantitative analysis of four flavonols in 109 hawk tea samples from various regions. Through transcriptome association analysis, SNPs linked to the biosynthesis of four flavonol glycosides were identified within the hawk tea transcriptome. This discovery lays the groundwork for future efforts to pinpoint genes related to hawk tea.

Initially, a population consisting of 109 individual trees from five regions in Guizhou, China, was constructed for the study. Through deep sequencing, each sample exhibited over 600,000 SNPs (Figure 5), indicating high genetic diversity within this group. Transcriptome association analysis revealed a set of candidate genes related to the content of four types of flavonols. Based on the correction for multiple testing and setting the *p* value threshold at *p* < 0.0001, 13 SNPs were identified as significant for functional annotation (Table 3). Functional annotation showed that these genes mainly belong to categories such as metabolic pathways, biosynthesis of secondary metabolites, and transport of secondary metabolites. Notably, among the candidate genes associated with K‐3‐O‐*β*‐D‐gal, one was annotated as UGT74B1. Jiang et al. (2018) found that *UGT* genes might be related to the biosynthesis of K‐3‐O‐*β*‐D‐gal and K‐3‐O‐*β*‐D‐glu, while Zhang et al. (2021) found that Q‐3‐O‐*β*‐D‐glu has a certain inhibitory effect on recombinant *UGT1A* subtypes in vitro. Moreover, as indicated by the data presented in Table 3, the structural genes (cytochrome P450 enzyme, selenium‐binding protein, glycoside glycosyltransferase, phosphoenolpyruvate carboxylase, and diacylglycerol acyltransferase) were found to be directly engaged in established pathways governing flavonoid metabolism, thus holding pivotal significance in flavonol biosynthesis. It is worth mentioning that the cytochrome P450 (CYP) family, which is annotated in both K‐3‐O‐*β*‐D‐Gal and K‐3‐O‐*β*‐D‐Glu, is a complex and widely distributed family whose members are involved in the synthesis of metabolites (Table 3) (Li et al. 2024). In addition, the interaction of flavonoids with cytochrome enzymes may prevent diseases (Karancsi et al. 2023). CYP4 and microsome could block 20‐hydroxyeicosatetraenoic acid (HETE) formation, which is beneficial to human health (Steuck, Hellhake, and Schebb 2016). The *CYP19* gene encodes aromatase, dietary flavonoids could inhibit the expression of CYP19 aromatase and play an auxiliary role in the prevention of estrogen diseases (Recalde‐Gil et al. 2019). Inhibition of CYP26 enzyme may prolong the half‐life of retinoic acid catabolism and provide a way for the treatment of related diseases (Lepri et al. 2018).

A natural population comprising 109 samples characterized by a limited diversity of samples from various regions and possessing a relatively complex structure impacted the outcomes of the association analysis, generally yielding a low association signal. Nonetheless, the considerable sequencing depth and comprehensive transcriptome coverage achieved in this study, coupled with the high density and reliability of the identified loci within the transcriptome, safeguarded the accuracy of the association signals.

Although candidate genes associated with flavonol content were not further analyzed and verified in this study, it represents the inaugural effort to perform an association analysis of hawk tea at the transcriptome level. This pioneering research holds significant implications for advancing our understanding of the genes and genetic mechanisms underlying the important secondary metabolites in hawk tea.

## 5 Conclusions

To summarize, results reveal no significant regional variation in DBH in hawk tea across Guizhou, highlighting that the diversity in leaf traits and flavonol levels primarily originates from habitat differences. Flavonol content emerged as a crucial determinant of hawk tea taste, exhibiting a notable correlation with tree age. Leaves of superior quality, distinguished by their flavonol levels, proved optimal for hawk tea production. Integrating second‐ and third‐generation transcriptome sequencing technologies enhances the generation of high‐quality transcripts, proving to be an efficacious strategy. Through transcriptome association analysis, 13 significant SNPs were identified to link to flavonol content, situated within gene regions. Notably, structural genes (including cytochrome P450 enzyme, selenium‐binding protein, glycoside glycosyltransferase, phosphoenolpyruvate carboxylase, and diacylglycerol acyltransferase) were pointed as integral components of known pathways directly regulating flavonoid metabolism and playing pivotal roles in flavonol biosynthesis. The findings lay a robust theoretical groundwork for the subsequent implementation of effective selection and breeding strategies in hawk tea.

## Author Contributions

**Lan Yang:** conceptualization (equal), data curation (equal), formal analysis (equal), methodology (equal), software (equal), validation (equal), writing – original draft (equal), writing – review and editing (equal). **Huie Li:** conceptualization (equal), validation (equal), writing – original draft (equal), writing – review and editing (equal). **Na Xie:** data curation (equal), formal analysis (equal), investigation (equal), methodology (equal), resources (equal), software (equal), validation (equal). **Gangyi Yuan:** data curation (equal), formal analysis (equal), investigation (equal), methodology (equal), resources (equal), software (equal), validation (equal). **Qiqiang Guo:** conceptualization (equal), data curation (equal), formal analysis (equal), funding acquisition (equal), investigation (equal), methodology (equal), resources (equal), software (equal), validation (equal), writing – original draft (equal), writing – review and editing (equal).

## Conflicts of Interest

The authors declare no conflicts of interest.

## Supporting information


Table S1.

## Data Availability

Raw reads have been deposited in the National Center for Biotechnology Information (NCBI; BioProject accession number PRJNA992466, https://www.ncbi.nlm.nih.gov/bioproject/?term=PRJNA992466).
