# Supplementary material for: Transcriptome‐Wide Association Analysis of Flavonoid Biosynthesis Genes and Their Correlation With Leaf Phenotypes in Hawk Tea ( Litsea coreana var. sinensis )
Source: Ecol Evol. 2024 Nov 17;14(11):e70563. doi: 10.1002/ece3.70563 (PMC11569866; doi:10.1002/ece3.70563)
Supplement: Supplementary file 1 — Table S1. [file ECE3-14-e70563-s001.doc]

**Table S1.** Significant markers and functional annotations of flavonol-related traits

| Flavonol | Chromosome | SNP number | Interval position | Significant SNP position | Allele type | P valuae | -log10 P valuae | Variant type | Annotation |
| --- | --- | --- | --- | --- | --- | --- | --- | --- | --- |
| K-3-O-β-D-Gal | CM022944.1 | 3 | 135222407-135422312 | 135322385 | T/C | 1.06E-08 | 7.974694135 | synonymous | - |
|  | CM022944.1 | 2 | 121837163-122036806 | 121937053 | A/G | 6.43E-08 | 7.191789027 | intron | Cytochrome P450 CYP4/CYP19/CYP26 subfamilies |
|  | CM022944.1 | 4 | 135231037-135430951 | 135330955 | C/G | 4.05E-07 | 6.392544977 | missense | Iron/ascorbate family oxidoreductases |
|  | CM022945.1 | 6 | 1457099-1642870 | 1556620 | C/G | 2.41E-08 | 7.617982957 | missense | Apoptotic ATPase |
|  | CM022946.1 | 2 | 20350230-20534664 | 20445096 | A/C | 6.91E-07 | 6.160521953 | synonymous | Uncharacterized conserved protein |
|  | CM022946.1 | 10 | 13099893-13291639 | 13199858 | T/C | 9.70E-07 | 6.013228266 | downstream gene | Selenium-binding protein |
|  | CM022946.1 | 1 | 693669-878016 | 786853 | G/A | 9.95E-07 | 6.002176919 | missense | WD40 repeat protein |
|  | CM022951.1 | 3 | 45903969-46103303 | 46003306 | T/A | 1.33E-07 | 6.876148359 | missense | - |
|  | CM022951.1 | 1 | 4276866-4467617 | 4376770 | T/C | 5.66E-07 | 6.247183569 | intergenic region |  |
|  | CM022952.1 | 1 | 25459077-25639627 | 25558941 | T/C | 1.88E-07 | 6.725842151 | missense | UDP-glucuronosyl and UDP-glucosyl transferase |
|  | CM022954.1 | 1 | 702550-885198 | 791508 | G/A | 7.27E-07 | 6.138465589 | intergenic region |  |
| K3-O-β-D-Glu | CM022944.1 | 3 | 158444114-158634618 | 158536066 | T/C | 2.74E-07 | 6.562446271 | missense | - |
|  | CM022944.1 | 2 | 121837163-122036806 | 121937053 | A/G | 6.60E-07 | 6.180272647 | intron | Cytochrome P450 CYP4/CYP19/CYP26 subfamilies |
|  | CM022945.1 | 5 | 99933346-100131398 | 100031720 | C/G | 7.71E-07 | 6.11272628 | missense | Ca2+-independent phospholipase A2 |
|  | CM022947.1 | 2 | 115382619-115576653 | 115476661 | C/A | 8.60E-07 | 6.065618045 | downstream gene | - |
|  | CM022948.1 | 1 | 97827104-98025021 | 97926933 | T/A | 8.24E-07 | 6.083821224 | intergenic region |  |
|  | CM022949.1 | 1 | 78896781-79089239 | 78996781 | T/C | 9.82E-07 | 6.007964022 | intergenic region |  |
|  | CM022950.1 | 8 | 67774743-67974291 | 67874309 | C/G | 4.01E-07 | 6.397167827 | missense&splice region | WD40 repeat nucleolar protein Bop1, involved in ribosome biogenesis |
| Q-3-O-β-D-Gal | CM022944.1 | 9 | 11393080-11583680 | 11492543 | T/C | 5.04E-08 | 7.29748671 | upstream gene | Cyclophilin type peptidyl-prolyl cis-trans isomerase |
|  | CM022944.1 | 1 | 9686869-9883095 | 9784107 | T/G | 1.26E-07 | 6.898858144 | intron | - |
|  | CM022944.1 | 5 | 140492328-140684317 | 140592264 | C/T | 3.82E-07 | 6.417936637 | splice donor&intron | Voltage-gated shaker-like K+ channel, subunit beta/KCNAB |
|  | CM022944.1 | 2 | 7352452-7552145 | 7452255 | A/T | 6.71E-07 | 6.172988403 | upstream gene | - |
|  | CM022945.1 | 3 | 39581062-39779313 | 39681060 | G/A | 8.80E-08 | 7.055644045 | intron | Predicted importin 9 |
|  | CM022945.1 | 2 | 45036521-45236437 | 45136445 | G/T | 3.55E-07 | 6.449463801 | intron | CCAAT-binding factor, subunit A (HAP3) |
|  | CM022945.1 | 2 | 28037050-28234964 | 28136888 | A/G | 4.30E-07 | 6.366688887 | intron | Serine/threonine protein phosphatase 2A, regulatory subunit |
|  | CM022945.1 | 4 | 97294227-97493288 | 97394210 | G/A | 5.91E-07 | 6.228231067 | upstream gene | - |
|  | CM022945.1 | 1 | 49598158-49790711 | 49690738 | C/T | 6.58E-07 | 6.181861124 | intron | AAA+-type ATPase containing the peptidase M41 domain |
|  | CM022945.1 | 14 | 16851515-17051392 | 16951424 | T/C | 8.58E-07 | 6.066544126 | downstream gene | Calcium transporting ATPase |
|  | CM022945.1 | 1 | 119092919-119284916 | 119184957 | G/A | 8.82E-07 | 6.054720889 | intergenic region |  |
|  | CM022946.1 | 3 | 112924717-113099887 | 113019610 | T/C | 3.65E-08 | 7.437631979 | downstream gene | Protein required for fusion of vesicles in vesicular transport, alpha-SNAP |
|  | CM022946.1 | 1 | 129956555-130152255 | 130056361 | C/G | 1.80E-07 | 6.744509699 | intergenic region |  |
|  | CM022947.1 | 3 | 118136800-118332146 | 118236785 | T/A | 5.10E-08 | 7.29275854 | upstream gene | UDP-galactose transporter related protein |
|  | CM022947.1 | 4 | 124993523-125181980 | 125082013 | C/T | 8.72E-08 | 7.059695821 | intron | Transcription factor MEIS1 and related HOX domain proteins |
|  | CM022947.1 | 2 | 94077372-94277302 | 94177329 | C/T | 1.38E-07 | 6.859995229 | downstream gene | - |
|  | CM022947.1 | 3 | 124860151-125038402 | 124944247 | T/C | 1.78E-07 | 6.748816551 | upstream gene | Mitogen-activated protein kinase |
|  | CM022947.1 | 1 | 109489925-109689874 | 109589901 | A/G | 4.13E-07 | 6.384350912 | synonymous | FOG: Leucine rich repeat |
|  | CM022947.1 | 2 | 7503400-7695648 | 7598503 | C/T | 4.89E-07 | 6.311113136 | intron | Dihydrolipoamide acetyltransferase |
|  | CM022947.1 | 1 | 37754410-37954360 | 37854393 | T/C | 8.53E-07 | 6.068988761 | intergenic region |  |
|  | CM022948.1 | 1 | 76327368-76514374 | 76424206 | G/T | 2.53E-08 | 7.596738733 | intergenic region |  |
|  | CM022948.1 | 1 | 23944418-24142960 | 24043698 | C/T | 9.43E-07 | 6.025476624 | intergenic region |  |
|  | CM022948.1 | 2 | 20120891-20299257 | 20220013 | A/G | 9.83E-07 | 6.007419986 | upstream gene | - |
|  | CM022949.1 | 4 | 23702427-23901588 | 23801610 | G/A | 4.95E-08 | 7.305211636 | missense | - |
|  | CM022949.1 | 7 | 19448064-19645155 | 19548052 | A/G | 3.97E-07 | 6.401386113 | upstream gene | Cytosolic sorting protein GGA2/TOM1 |
|  | CM022949.1 | 1 | 5161220-5347868 | 5253374 | A/C | 5.95E-07 | 6.225784925 | intergenic region |  |
|  | CM022949.1 | 5 | 19093858-19283923 | 19186355 | A/G | 8.06E-07 | 6.093773159 | intron | Nucleotide excision repair factor NEF2, RAD4/CUT5 component |
|  | CM022949.1 | 1 | 15057934-15254602 | 15156255 | A/G | 8.84E-07 | 6.053727385 | intron | - |
|  | CM022950.1 | 6 | 81467991-81658800 | 81567923 | C/T | 1.75E-07 | 6.756020265 | intron | Serine/threonine protein kinase |
|  | CM022950.1 | 2 | 5370951-5564146 | 5464174 | A/T | 1.93E-07 | 6.715463721 | downstream gene | - |
|  | CM022951.1 | 1 | 64927876-65127435 | 65027452 | G/A | 2.13E-08 | 7.672591481 | intron | Mitogen-activated protein kinase |
|  | CM022952.1 | 2 | 6050076-6235197 | 6149842 | A/T | 9.38E-07 | 6.027925479 | downstream gene | - |
|  | CM022953.1 | 1 | 64339010-64520493 | 64420900 | T/C | 8.51E-08 | 7.070214584 | intergenic region |  |
|  | CM022954.1 | 5 | 58798027-58993207 | 58893692 | G/A | 2.69E-08 | 7.570174014 | intron | RNA polymerase II transcription mediator |
|  | CM022955.1 | 1 | 12176601-12376468 | 12276483 | A/G | 8.14E-07 | 6.089474724 | intergenic region |  |
| Q-3-O-β-D-Glu | CM022949.1 | 1 | 76530466-76715668 | 76615725 | A/C | 6.65E-09 | 8.176971323 | intergenic region |  |
|  | CM022949.1 | 3 | 76511041-76710872 | 76610892 | G/A | 7.41E-09 | 8.130181792 | downstream gene | - |
|  | CM022945.1 | 1 | 125049876-125244193 | 125146383 | G/A | 3.55E-08 | 7.449771647 | missense | Mitochondrial transcription termination factor, mTERF |
|  | CM022944.1 | 2 | 127993307-128193207 | 128093232 | G/T | 5.81E-08 | 7.235823868 | upstream gene | - |
|  | CM022948.1 | 3 | 20120891-20299257 | 20220013 | A/G | 6.11E-08 | 7.21395879 | splice region&intron | - |
|  | CM022953.1 | 1 | 47392713-47569122 | 47477583 | T/C | 7.88E-08 | 7.103473783 | upstream gene | Scaffold/matrix specific factor hnRNP-U/SAF-A, contains SPRY domain |
|  | CM022947.1 | 1 | 2465507-2657954 | 2558160 | C/T | 8.37E-08 | 7.077274542 | intergenic region |  |
|  | CM022951.1 | 1 | 4276545-4467617 | 4370334 | A/G | 9.13E-08 | 7.039529222 | missense | WD40-repeat-containing subunit of the 18S rRNA processing complex |
|  | CM022948.1 | 2 | 56870506-57068785 | 56968787 | A/C | 9.17E-08 | 7.037630664 | synonymous | - |
|  | CM022945.1 | 2 | 33837640-34028492 | 33929352 | T/A | 9.32E-08 | 7.030584088 | splice donor&intron | Prolyl 4-hydroxylase alpha subunit |
|  | CM022955.1 | 3 | 8613233-8794085 | 8708530 | G/A | 1.04E-07 | 6.982966661 | missense | - |
|  | CM022947.1 | 6 | 131454061-131649671 | 131554058 | T/C | 1.27E-07 | 6.896196279 | synonymous | - |
|  | CM022946.1 | 1 | 12177781-12370848 | 12277330 | G/T | 1.45E-07 | 6.838631998 | synonymous | Sterol O-acyltransferase/Diacylglycerol O-acyltransferase |
|  | CM022947.1 | 3 | 7430188-7629881 | 7529974 | T/A | 1.63E-07 | 6.787812396 | downstream gene | - |
|  | CM022952.1 | 2 | 6317609-6495002 | 6417586 | T/C | 2.40E-07 | 6.619788758 | intron | Transcription factor MEIS1 and related HOX domain proteins |
|  | CM022952.1 | 2 | 6317609-6495002 | 6417587 | G/A | 2.40E-07 | 6.619788758 | intron | Lecithin:cholesterol acyltransferase (LCAT)/Acyl-ceramide synthase |
|  | CM022946.1 | 1 | 112873217-113052890 | 112964709 | C/T | 2.58E-07 | 6.588380294 | intron | 60s ribosomal protein L34 |
|  | CM022954.1 | 5 | 58798027-58993207 | 58893692 | G/A | 3.23E-07 | 6.490797478 | upstream gene | - |
|  | CM022946.1 | 3 | 4857410-5047966 | 4956886 | G/A | 3.74E-07 | 6.427128398 | intergenic region |  |
|  | CM022951.1 | 1 | 64927876-65127435 | 65027452 | G/A | 4.20E-07 | 6.37675071 | synonymous | Isoamyl acetate-hydrolyzing esterase and related enzymes |
|  | CM022944.1 | 3 | 109960797-110151955 | 110053148 | A/T | 4.65E-07 | 6.332547047 | missense | Isoamyl acetate-hydrolyzing esterase and related enzymes |
|  | CM022953.1 | 2 | 8597276-8794356 | 8695728 | T/C | 7.23E-07 | 6.140861703 | intron | Predicted DHHC-type Zn-finger protein |
|  | CM022947.1 | 2 | 140132680-140332146 | 140232643 | G/T | 7.36E-07 | 6.133122186 | intergenic region |  |
|  | CM022950.1 | 1 | 79796559-79987992 | 79888055 | A/G | 7.99E-07 | 6.097453221 | intron | Mitogen-activated protein kinase |
|  | CM022947.1 | 2 | 94077372-94277302 | 94177329 | C/T | 8.13E-07 | 6.089909454 | synonymous | FOG: PPR repeat |
|  | CM022946.1 | 4 | 10337994-10522724 | 10433828 | C/T | 8.69E-07 | 6.060980224 | missense | FOG: PPR repeat |
|  | CM022945.1 | 4 | 113462692-113662076 | 113562078 | A/C | 9.02E-07 | 6.044793462 | synonymous | SNF2 family DNA-dependent ATPase |
|  | CM022944.1 | 6 | 12619168-12814254 | 12714282 | A/G | 9.57E-07 | 6.019088062 | intergenic region |  |
|  | CM022947.1 | 4 | 124993523-125181980 | 125082013 | C/T | 9.61E-07 | 6.017276612 | intron |  |
|  | CM022945.1 | 3 | 26125074-26321333 | 26223647 | C/A | 9.75E-07 | 6.010995384 | missense | Apoptotic ATPase |
